# Real-World Evaluation of the Episoft® AC Moisturizer With Broad-Spectrum Sunscreen in the Management of Retinoid-Induced Dermatitis: A Retrospective Observational Study

**DOI:** 10.7759/cureus.108616

**Published:** 2026-05-10

**Authors:** Gaurav Nakra, Ashwini Modi, Swetha Hegde, Sanchaita Bala, Sumit Bhushan, Dhiraj Dhoot, Ashwin Balasubramanian, Prajakta Bhosale, Rujuta Gadkari, Saiprasad Patil

**Affiliations:** 1 Dermatology, Centre for Skin, New Delhi, IND; 2 Dermatology, Elevé Skin Clinic, Mumbai, IND; 3 Dermatology, Remedial Rays Skin Care Clinic, Bengaluru, IND; 4 Dermatology, Remedy Diagnostics, Kolkata, IND; 5 Global Medical Affairs, Glenmark Pharmaceuticals Limited, Mumbai, IND; 6 Dermatology, Glenmark Pharmaceuticals Limited, Mumbai, IND; 7 Global Medial Affairs, Glenmark Pharmaceuticals Limited, Mumbai, IND

**Keywords:** acne, adherence, barrier repair, moisturizer-sunscreen combination, photoprotection, real-world evidence, retinoid-induced dermatitis, skin barrier dysfunction, tolerability, transepidermal water loss

## Abstract

Background

Retinoids are essential in acne management but frequently cause retinoid‑induced dermatitis (RID), characterized by erythema, dryness, burning, and barrier disruption. This study evaluated the real‑world effectiveness and tolerability of Episoft® AC (Glenmark Pharmaceuticals, Mumbai, India), a fixed-dose combination moisturizer with broad‑spectrum sunscreen, in patients experiencing RID.

Methods

This study was a retrospective, multicentric observational analysis of medical records of acne patients with RID, conducted across 357 dermatology clinics in India, following an independent ethics committee approval (approval no. GSER/2024/BMR-AP/133). The study included 1364 patients who had been prescribed Episoft® AC for managing RID. The mean age was 24.49 years (SD ±5.16), and both male and female participants were included. Subjective symptoms (itching, burning, stinging, tightness) and objective signs (erythema, dark spots, dryness, roughness) were assessed at baseline, Week 2, and Week 4 using a four‑point Likert severity scale. Corneometer‑based skin hydration and adverse events (AEs) were also evaluated. The primary endpoint was reduction in dermatitis symptoms; secondary endpoints were Corneometer-measured skin hydration and overall tolerability.

Results

A total of 1039 (76%) patients were prescribed a topical retinoid or combination, most commonly 0.1% adapalene + 2.5% benzoyl peroxide (BPO), and 294 (22%) were prescribed oral isotretinoin. Significant improvement was observed across all parameters. By Week 4, complete resolution was reported by 68.7% of patients for itching, by 88.44% for burning, by 69.9% patients for stinging, by 72.63% for tightness, and by 61.69% patients for erythema. In addition, physicians observed significant improvement in dark spots (74.42%), dryness (70.59%), and roughness (73.73%). Hydration improved by 39.91% on Corneometer assessment. AEs were considered predominantly retinoid-associated based on clinical context; definitive causality attribution was not feasible given the retrospective, single-arm, uncontrolled design. Only 1.17% reported mostly mild‑to‑moderate AEs, which were associated with retinoid use.

Conclusion

Episoft® AC substantially improved the tolerance of topical and systemic retinoid therapy, reducing dermatitis symptoms, enhancing skin hydration, and demonstrating excellent safety. It represents a practical adjunct to support adherence and mitigate RID in routine dermatology practice.

## Introduction

Retinoids remain a cornerstone in the management of acne vulgaris and photoaging due to their ability to normalize keratinocyte differentiation and stimulate dermal remodelling [1,2]. However, their use is often limited by retinoid‑induced dermatitis (RID), a predictable constellation of erythema, dryness, burning, and barrier disruption that reduces adherence and compromises therapeutic outcomes [3,4]. RID arises primarily from stratum corneum dysfunction, increased transepidermal water loss (TEWL), and inflammation following initiation of retinoid therapy [5].

Histologic and clinical improvement in sun-exposed skin following topical retinoic acid treatment is well documented. However, daily application of retinoic acid commonly induces an erythematous, scaling reaction that appears within two to five days and typically diminishes with continued use. The underlying cellular, immunologic, and biochemical mechanisms of this retinoid-induced reaction, as well as its relationship with the repair of photodamaged skin, remain incompletely understood [6].

Recent studies, such as that by Fang et al. (2024), have demonstrated that physiologic lipids, particularly phytosteryl/octyldodecyl lauroyl glutamate (PLG), effectively mitigate retinol-induced irritation, providing a strong rationale for incorporating lipid-based solutions into retinoid formulations [7].

A well-established approach to mitigating these side effects is the concurrent use of moisturizers and sunscreens, which help to restore hydration, reduce irritation, and protect against environmental damage. Moisturizers play a crucial role in enhancing skin hydration, reinforcing barrier function, and minimizing peeling, while sunscreens prevent ultraviolet (UV)-induced exacerbation of irritation and post-inflammatory hyperpigmentation, and are crucial to prevent and alleviate RID, enhance treatment tolerability, reduce photodermatitis, and improve patient adherence [8-10]. However, despite being widely recommended, the separate application of these products may not always ensure consistent adherence, leading to suboptimal skin barrier repair and prolonged discomfort [11].

While prior studies have evaluated moisturizers and sunscreens as separate adjuncts in general acne management, there remains a specific and largely unaddressed clinical need for a single, fixed-dose combination product that simultaneously delivers moisturization and broad-spectrum photoprotection in patients experiencing retinoid-induced dermatitis. Separate application of these products in routine practice is associated with inconsistent adherence, suboptimal barrier repair, and prolonged patient discomfort - gaps that a combined formulation like Episoft® AC (Glenmark Pharmaceuticals, Mumbai, India) is uniquely positioned to address by simplifying the skincare regimen, reducing treatment burden, and supporting sustained retinoid use.

Despite the well-recognized role of moisturizers and sunscreens as adjuncts in retinoid therapy, real-world evidence on fixed-dose moisturizer-sunscreen combinations specifically in patients experiencing RID remains limited. Existing studies have largely evaluated these agents separately or in broader acne populations, leaving a gap in evidence for their combined use in the specific clinical context of RID. Therefore, this study aimed to evaluate the real-world efficacy and safety of Episoft® AC in mitigating retinoid-induced dermatitis in routine dermatological practice.

Episoft® AC, a fixed-dose combination of a physiologic moisturiser and broad-spectrum sunscreen, contains an emollient plus humectant combination of capric triglycerides and D-panthenol, along with microencapsulated chemical sunscreen filters. Its formulation replenishes water content, restores lipid balance, and provides photoprotection, thereby preventing both barrier disruption and UV sensitivity inherent to retinoid therapy [12].

Building on prior evidence where Episoft® AC SPF 30 demonstrated meaningful improvement in facial skin tolerability among a large cohort of acne patients [12], the present study was designed to further evaluate its role in a clinically relevant population - patients experiencing retinoid‑induced dermatitis. Given the well‑recognized challenges of dryness, irritation, and barrier disruption associated with retinoid therapy, a formulation that combines moisturization with broad‑spectrum photoprotection offers a practical strategy to enhance comfort and support adherence.

## Materials and methods

This was a multicentric, retrospective, observational study conducted across 357 dermatology clinics in India. Data were extracted from the existing medical records of 1364 patients who had been prescribed Episoft® AC for the management of retinoid-induced dermatitis, over a four-week treatment period. As this was a retrospective analysis of anonymized data from routine clinical practice, individual patient consent was not required. The study adhered to the principles of the Declaration of Helsinki, complied with applicable Indian regulatory requirements (including the New Drugs and Clinical Trials Rules, 2019) [13], and received ethics committee approval (approval no. GSER/2024/BMR-AP/133, from the Good Society Ethical Research Independent Ethics Committee, dated August 31, 2024) before study initiation.

Eligible participants were males or females aged 12 years or older who had been clinically diagnosed with RID following treatment for acne vulgaris and were prescribed Episoft® AC by their dermatologist. Patients receiving either topical or systemic retinoid therapy and maintaining regular follow-up during the observation period were included. Follow-up data were extracted from available medical records at Weeks 2 and 4 for all eligible patients. Patients with incomplete records at any assessment time point were not excluded from the overall analysis but were accounted for by reporting the actual number of patients assessed at each visit for each endpoint separately. No imputation of missing data was performed, and analyses were conducted on available case data only. The variable denominators reported across endpoints reflect the real-world nature of the retrospective data collection, wherein not all symptoms and signs were present or documented for every patient at every visit.

Patients with severe (presence of nodules and cysts) acne, those who modified their anti-acne regimen during the study period, or individuals who underwent concurrent dermatological procedures such as chemical peels or laser treatments, and those with incomplete medical records were excluded. Patients with other visible dermatologic disorders or abnormal skin pigmentation that may have interfered with subjective or objective assessments were also excluded.

The primary endpoint was the reduction in severity of retinoid-induced dermatitis (categorized as mild, moderate, or severe) from baseline to Week 4, evaluated using both subjective (patient-reported) and objective (dermatologist-assessed) measures. Secondary endpoints included the incidence of adverse events (AEs) associated with the use of Episoft® AC during the observation period. Corneometer-based skin hydration assessment was performed as an exploratory endpoint in a subset of patients at centers where the device was available. Measurements were obtained on the facial skin following a standardized acclimatization period, under controlled ambient conditions, with patients instructed to refrain from applying any topical skin products for a minimum of two hours before assessment. The subset of patients undergoing Corneometer assessment (n=162 at baseline; n=158 at Weeks 2 and 4) was not randomly selected but represented those attending centers equipped with Corneometer devices. This constituted an exploratory, convenience-based subset, and the findings should therefore be interpreted as supportive and hypothesis-generating rather than definitive. An exploratory endpoint was the percentage increase in skin hydration, measured using Corneometer® in as many patients as possible.

Corneometer readings are reported in arbitrary units (AU) as generated by the device following internal calibration adjustment. The negative minimum values observed at baseline (min.: -2 AU) and Week 4 (min.: -4 AU) represent calibration-adjusted outputs at the lower sensitivity threshold of the instrument and do not indicate physiologically impossible hydration states. These values reflect extreme inter-individual variability in baseline skin hydration, particularly in patients with significantly compromised barrier function secondary to retinoid-induced dermatitis, and are consistent with previously reported real-world Corneometer datasets in dermatologically compromised skin.

RID was diagnosed clinically by qualified dermatologists based on the temporal relationship between retinoid therapy initiation and the onset of characteristic cutaneous signs and symptoms. The diagnosis was established when patients presented with one or more of the following features within the expected timeframe of retinoid exposure: erythema, dryness, roughness, scaling, burning, stinging, itching, and skin tightness, occurring in the context of ongoing topical or systemic retinoid therapy for acne vulgaris, in the absence of other confounding dermatological conditions.

To ensure consistency of data collection across all 357 participating centers, a standardized, predesigned electronic case report form was developed and uniformly implemented to collect and extract demographic information, retinoid-induced dermatitis symptoms from baseline to Week 4, and adverse events.

For subjective assessment, data regarding various symptoms such as itching, burning, stinging, and skin tightness experienced by the patients during the previous eight weeks were analysed. Participating dermatologists received structured guidance on clinical assessment criteria, including the definitions and grading of retinoid-induced dermatitis symptoms using the validated four-point Likert severity scale (0-3), before study initiation. Objective parameters, including erythema, dryness, roughness, and dark spots, were assessed using predefined clinical descriptors to minimize inter-observer variability. For objective assessment, data regarding visible signs such as erythema, dark spots, dryness, and roughness evaluated by the investigators during the previous four weeks were analysed. Each parameter was measured on a four-point Likert scale (0-3) [14] ranging from no evidence of any facial skin symptom to severe facial skin symptom (none, mild, moderate, severe). The parameters were evaluated at baseline, Week 2, and Week 4.

In addition to descriptive statistics, inferential statistical analyses were performed to evaluate change over time. For subjective and objective Likert-scale outcomes, the Wilcoxon signed-rank test was applied to assess paired comparisons between baseline and Week 2, and baseline and Week 4, given the ordinal nature of the data. For Corneometer-based continuous hydration values, a paired t-test was used to evaluate mean change from baseline to Week 2 and Week 4. All analyses were two-tailed with a significance threshold of p<0.05. Results are presented with 95% confidence intervals (CIs) where applicable. Data were summarized using descriptive statistics and are presented as n (%) for categorical variables and means ± SDs, medians, for continuous variables.

A sensitivity analysis was performed stratifying the cohort by retinoid class and regimen to evaluate whether the observed improvements in RID endpoints were consistent across materially different treatment contexts. Stratified analyses were conducted for the following groups: adapalene monotherapy, adapalene combined with benzoyl peroxide, adapalene combined with clindamycin, tretinoin, other topical retinoids, and oral isotretinoin. The direction and magnitude of improvement across all primary endpoints remained broadly consistent across all retinoid subgroups, supporting the generalizability of the overall findings while acknowledging important between-group differences in absolute resolution rates and adverse event profiles.

## Results

Demographic and baseline characteristics

A total of 1364 medical records were eligible for analysis. The mean age of the population was 24.49 years (SD ±5.16). In terms of gender distribution, 575 participants (42.15%) were male, whereas 789 participants (57.85%) were female. The most frequently used treatment was adapalene combined with benzoyl peroxide (361 cases, 26.47%), followed by isotretinoin (294 cases, 21.55%) and adapalene monotherapy (286 cases, 20.97%). Detailed demographic and baseline characteristics are given in Tables 1-3.

**Table 1:** Demographic and baseline characteristics

Parameters		No. of cases (N)	Mean ± SD/percentage
Age (years)		1364	24.49 ± 5.16
Sex	Male	575	42.15%
	Female	789	57.85%

**Table 2:** Prevalence of acne signs and symptoms

Category	Signs/symptoms	N	Percentage
Subjective (patient reported)	Itching	898	65.84%
	Burning	554	40.62%
	Stinging	874	64.08%
	Skin tightness	866	63.49%
Objective (dermatologist assessed)	Erythema	603	44.21%
	Dark spots	563	41.28%
	Dryness	925	67.82%
	Roughness	929	68.10%

**Table 3:** Distribution of anti-acne medical therapies used

Therapy	N	Percentage
Topical retinoid therapy		
Adapalene	286	20.97%
Adapalene + benzoyl peroxide	361	26.47%
Adapalene other combination	9	0.66%
Adapalene + clindamycin	155	11.36%
Other retinoid therapy	120	8.80%
Tretinoin	108	7.92%
Oral retinoid therapy		
Isotretinoin	294	21.55%
Topical antibiotic therapy		
Minocycline	20	1.47%
Clindamycin combination	6	0.44%
Clindamycin	5	0.37%

Symptom resolution from baseline to Week 4

**Figure 1:**
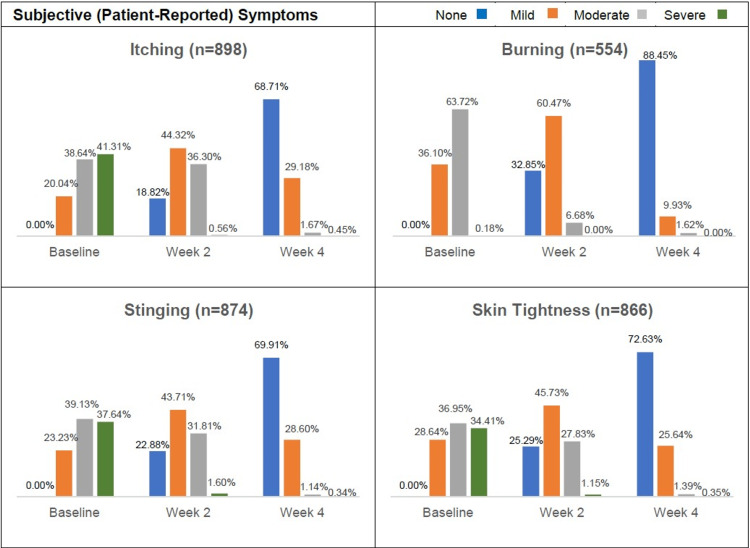
Improvement in subjective (patient reported) symptoms of retinoid-induced dermatitis observed at two and four weeks post-treatment initiation

**Figure 2:**
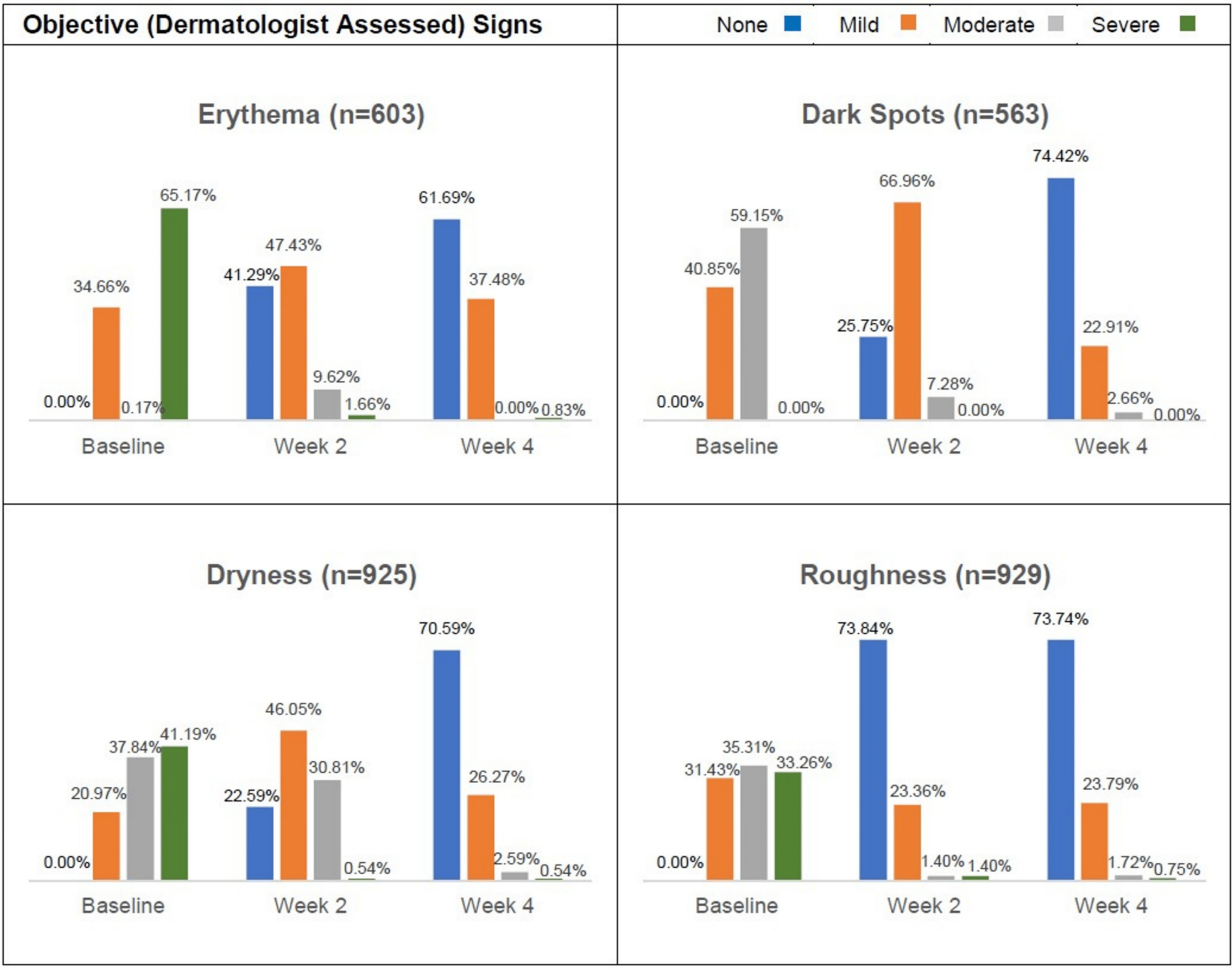
Improvement in objective (dermatologist assessed) signs of retinoid-induced dermatitis observed at two and four weeks post-treatment initiation

After four weeks, marked improvement was seen in signs and symptoms of retinoid-induced dermatitis as shown in Figures 1-2.

**Figure 3:**
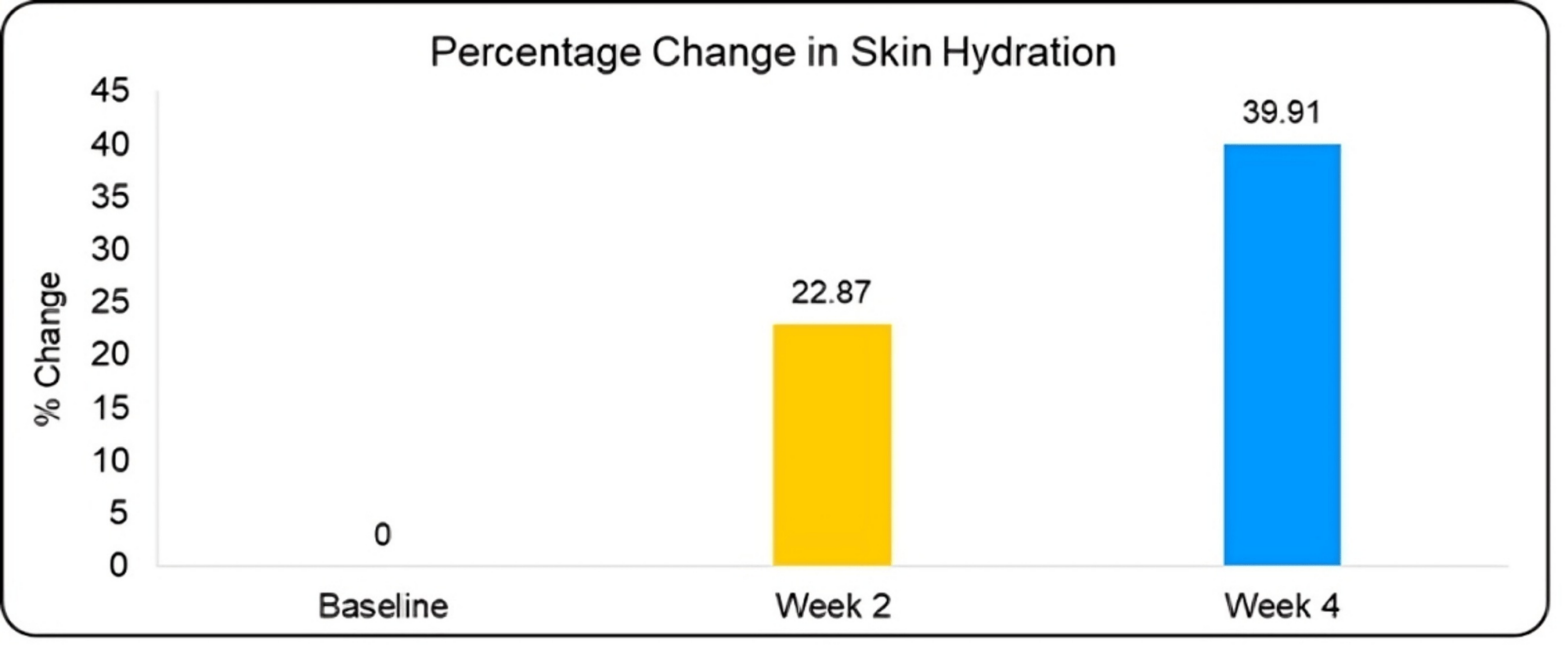
Percentage change in skin hydration from baseline observed at Weeks 2 and 4 post-treatment initiation

Skin hydration, measured using Corneometer readings, demonstrated a progressive improvement over the four-week study period, reflecting enhanced skin barrier function with almost a 23% improvement at Week 2 and a 40% improvement at Week 4 from baseline, as shown in Table 4 and Figure 3, reflecting a consistent and clinically meaningful improvement in skin hydration over time.

**Table 4:** Corneometer skin hydration readings over time

Visit	N	Statistics	Corneometer skin hydration reading (Arbitrary units – AU)
Baseline	162	Mean ± SD (min.:max.)	8.92 ± 13.11 (-2:40)
Week 2	158	Mean ± SD (min.:max.)	10.96 ± 13.95 (0:45)
Week 4	158	Mean ± SD (min.:max.)	12.48 ± 15.28 (-4:47)
Change from baseline to Week 2	158	Mean ± SD	2.04 ± 0.84
Change from baseline to Week 4	158	Mean ± SD	3.56 ± 2.17

Safety was evaluated based on the incidence, severity, and outcomes of AEs over the four‑week period. Overall, the formulation was well tolerated, with AEs occurring in only a small proportion of subjects. Most AEs were cutaneous in nature, with mild-to-moderate severity, and reflected expected retinoid‑associated reactions such as dryness, cheilitis, and photosensitivity. These findings are displayed in Table 5.

**Table 5:** Cumulative adverse events at the end of Week 4 MedDRA: Medical Dictionary for Regulatory Activities

MedDRA preferred term	No. of events, n (%)	No. of patients, n (%)	Same patient, multiple events
Acne	1 (0.07%)	1 (0.07%)	No
Cheilitis	6 (0.44%)	5 (0.37%)	Yes (1 patient)
Skin dryness	7 (0.51%)	6 (0.44%)	Yes (1 patient)
Exfoliation	2 (0.15%)	2 (0.15%)	No
Hyperhidrosis	1 (0.07%)	1 (0.07%)	No
Inflammatory papules and pustules	1 (0.07%)	1 (0.07%)	No
Skin irritation	2 (0.15%)	2 (0.15%)	No
Photosensitivity reaction	5 (0.37%)	5 (0.37%)	No
Erythema	1 (0.07%)	1 (0.07%)	No
Rosacea	1 (0.07%)	1 (0.07%)	No
Total	28 (2.05%)	16 (1.17%)	

## Discussion

In this retrospective observational study involving 1364 subjects, use of Episoft® AC was associated with clinically meaningful improvement in both subjective patient-reported and objective dermatologist-assessed RID symptoms over four weeks, with a large proportion of patients showing complete resolution or notable reduction in symptom severity. Given the uncontrolled single-arm design, these findings reflect real-world associations and should not be interpreted as evidence of definitive causal efficacy. Key subjective outcomes included high resolution rates by Week 4 for itching (68.7%), burning (88.44%), stinging (69.90%), and skin tightness (72.63%), alongside progressive reductions in severity starting as early as Week 2. Objective signs also showed significant reductions, with roughness (73.73%), dryness (70.59%), dark spots (74.42%), and erythema (61.69%) showing substantial clearance or downgrading to mild. Skin hydration also improved meaningfully, as Corneometer® hydration scores improved by 39.91% by Week 4. These real-world observations suggest that Episoft® AC may serve as a potentially useful adjunct to support adherence and alleviate RID-associated discomfort in routine dermatology practice. However, given the retrospective uncontrolled design and absence of a comparator group, recommendations for routine standard integration await confirmation from prospective comparative studies. Safety was excellent, with a low treatment emergent adverse event rate: 1.17% of subjects reporting 28 events, mostly mild-to-moderate retinoid-associated reactions like cheilitis, dryness, and photosensitivity that tended to resolve or lessen over time.

Retinoid therapy commonly disrupts the stratum corneum, increasing TEWL and leading to dryness, roughness, peeling, and irritation that collectively contribute to retinoid‑induced dermatitis. Restoring hydration is therefore central to maintaining barrier integrity and minimizing these effects. Adequate hydration stabilizes intercellular lipids and corneocytes, reduces inflammation, prevents excessive desquamation, and alleviates sensory symptoms such as stinging and burning. Moisturizers support this process through humectants that attract and retain water (e.g., glycerin, hyaluronic acid), occlusives that limit TEWL (e.g., petrolatum), and emollients that fill barrier gaps and mimic natural moisturizing factors (e.g., ceramides), thereby improving comfort and enabling continued retinoid use.

Clinical evidence consistently shows that adjunctive moisturizers enhance the tolerability of retinoid‑based regimens. A physiologic lipid blend containing phytosteryl/octyldodecyl lauryl glutamate significantly reduced retinol‑induced irritation in controlled patch and in‑use trials. An adjuvant dermocosmetic regimen similarly decreased burning, itching, and peeling associated with retinoid-benzoyl peroxide therapy. Barrier‑strengthening formulations, such as a *Macrocystis pyrifera* ferment cream used with tretinoin, have demonstrated reductions in TEWL and clinical irritation [15]. Randomized controlled trials further validate these benefits: Hayashi and Kawashima reported improved adherence and fewer irritation‑related dropouts when a heparinoid moisturizer was initiated with adapalene [16], while Zeichner et al. showed that a ceramide‑containing moisturizer minimized dryness and irritation during combination therapy without compromising efficacy [17]. Collectively, these findings confirm that moisturizers play a critical role in improving tolerability and supporting sustained retinoid use.

Sunscreens are a critical adjunct to retinoid therapy, as retinoids increase photosensitivity, thin the stratum corneum, and heighten vulnerability to UV‑induced irritation and post‑inflammatory hyperpigmentation. The broad-spectrum photoprotection provided by Episoft® AC was associated with observed reductions in photosensitivity-related complaints in this cohort, consistent with the well-recognized role of sunscreens in mitigating UV-exacerbated barrier damage during retinoid therapy. Whether this translates to meaningful prevention of post-inflammatory hyperpigmentation requires prospective evaluation with appropriate comparator groups. Major guidelines, including the 2024 American Academy of Dermatology Acne Guidelines, and recent Indian consensus statements (PRACT‑India; PRISM‑ISF), strongly recommend concurrent sunscreen use during retinoid therapy, with a preference for broad‑spectrum sunscreens in acne‑prone skin [8-10].

Evidence from randomized controlled trials further supports the importance of sunscreen as an adjunct to retinoid therapy. In a 2023 double‑blind RCT, How et al. showed that adding a sunscreen containing licochalcone A and L‑carnitine to adapalene significantly improved cutaneous tolerability, reducing dryness, scaliness, burning, and stinging, while also lowering post‑acne hyperpigmentation scores without affecting acne improvement [18]. Complementing this, Panda et al. demonstrated that a specially designed broad‑spectrum SPF 50+ sunscreen for retinol users provided high UV protection (critical wavelength >370 nm) along with anti‑irritant benefits, proving non‑irritant in patch testing and safe for sensitive, retinoid‑compromised skin [19]. Together, these findings highlight that appropriate sunscreen selection can meaningfully reduce irritation, prevent UV‑exacerbated damage, and enhance the overall tolerability of retinoid regimens.

Treatment adherence in dermatology is strongly influenced by regimen complexity, with Moradi Tuchayi et al. identifying “regimen too complex,” forgetfulness, inconvenience, and busy lifestyle as major secondary barriers contributing to nonadherence in acne management [11]. The authors emphasized that simplifying treatment steps, such as using combination products instead of multiple separate applications, is one of the most effective strategies to improve treatment adherence. In this context, a single formulation that integrates both moisturization and photoprotection can substantially reduce treatment burden, minimize the risk of under‑application, and support more consistent daily use.

Data from the study by Del Rosso et al. demonstrated that incorporating a skincare regimen consisting of a cleanser and a moisturizer with SPF 30 into adapalene-benzoyl peroxide therapy improved overall tolerability, reducing erythema, dryness, and stinging/burning, while enhancing patient satisfaction and maintaining adherence; importantly, the moisturizer-sunscreen formulation was non‑comedogenic and well tolerated [20]. Similarly, the CHARISMA study, which included 340 acne patients on stable therapy, showed that Episoft® AC consistently improved itching, burning, stinging, tightness, erythema, and dryness by Day 28, with benefits sustained through Day 56, alongside excellent tolerability, high adherence, and strong patient satisfaction [12]. Together, these findings support the role of non‑comedogenic moisturizer-sunscreen formulations as valuable adjuncts for improving comfort, adherence, and overall treatment experience in acne and retinoid‑associated skin irritation. The fixed-dose combination format of Episoft® AC was associated with observations suggesting reduced treatment burden and potentially improved adherence in this real-world cohort, consistent with the established literature on regimen simplification. However, formal adherence outcomes were not prospectively measured in this study, and these observations should be interpreted cautiously. Hence, the integration of a fixed-dose combination of moisturizer and sunscreen, like Episoft® AC, represents a convenient and evidence-based advancement in managing retinoid-induced dermatitis, directly aligning with established dermatological principles and clinical recommendations, addressing these needs in a single step, reducing regimen complexity, and supporting more consistent use, better tolerability, and sustained retinoid use and efficacy.

The present study demonstrated that Episoft® AC, a fixed-dose moisturizer-sunscreen combination, effectively managed retinoid-induced dermatitis, providing rapid relief from subjective symptoms (itching, burning, stinging, tightness) and objective signs (erythema, dryness, roughness, dark spots), with most patients achieving complete resolution or significant improvement by Week 4. Clinically, it enhanced retinoid tolerability and adherence by reducing irritation and photosensitivity in a single, convenient product. It supports proactive use from therapy initiation to prevent side effects and UV-exacerbated damage such as post-inflammatory hyperpigmentation, supporting its routine integration as a standard adjunct with retinoids for better patient compliance and long-term treatment success. The low adverse event rate of 1.17% observed in this cohort suggests a favorable tolerability profile for Episoft® AC in the real-world clinical setting. However, the absence of a comparator group and the retrospective nature of adverse event collection preclude definitive conclusions regarding comparative safety, and these findings should be interpreted within the constraints of the observational design.

The strengths of this study lie in its large sample size, comprehensive evaluation of subjective and objective outcomes, detailed longitudinal assessments at baseline, Week 2, and Week 4 showing rapid improvements, and a low adverse event rate supporting safety. Limitations include its single-arm, observational design, potential confounding from unstratified concomitant retinoid use, and a short four-week follow-up, lacking long-term data. While prospective and interventional, these factors moderate the evidence for definitive efficacy claims in managing retinoid-induced dermatitis. From a practical clinical perspective, the findings of this real-world study suggest that fixed-dose moisturizer-sunscreen combinations such as Episoft® AC may be considered as adjunctive support in specific patient populations who are at heightened risk of developing retinoid-induced dermatitis. These include patients initiating high-potency retinoid regimens such as oral isotretinoin or tretinoin, those with a prior history of sensitive or reactive skin, patients with darker phototypes who are at increased risk of post-inflammatory hyperpigmentation, adolescent patients with limited prior skincare experience, and those receiving combination retinoid regimens, such as adapalene with benzoyl peroxide, where additive irritancy potential is recognized.

Early proactive initiation of adjunctive moisturizer-sunscreen therapy from the commencement of retinoid treatment, rather than reactive use following established dermatitis, may offer greater benefit in terms of barrier preservation, symptom prevention, and sustained treatment adherence. These observations are hypothesis-generating and require confirmation from prospective comparative studies before formal clinical recommendations can be established.

## Conclusions

In this large real-world retrospective observational analysis conducted across 357 dermatology centers in India, use of Episoft® AC was associated with improvement in both patient-reported symptoms and dermatologist-assessed signs of retinoid-induced dermatitis over four weeks of routine clinical practice. Supportive improvements in skin hydration were observed in an exploratory subset of patients. The formulation was generally well tolerated, with a low rate of mostly mild-to-moderate adverse events predominantly attributable to concurrent retinoid therapy. These real-world observations are hypothesis generating and should not be interpreted as evidence of definitive causal efficacy or superiority over standard supportive care. Controlled prospective studies with appropriate comparator groups are needed to confirm these associations, establish comparative effectiveness, evaluate long-term durability, and support generalizable evidence-based recommendations for the routine clinical integration of fixed-dose moisturizer sunscreen combinations in the management of RID. These findings generate a clinically relevant hypothesis regarding the potential role of fixed-dose moisturizer-sunscreen combinations as adjuncts in RID management. Prospective randomized controlled studies with appropriate comparator groups are needed to confirm these associations, establish comparative effectiveness, and support evidence-based recommendations for routine clinical integration.
